# Pharmacological properties of baicalin on liver diseases: a narrative review

**DOI:** 10.1007/s43440-021-00227-1

**Published:** 2021-02-17

**Authors:** Jin-yu Yang, Min Li, Cheng-liang Zhang, Dong Liu

**Affiliations:** Department of Pharmacy, Tongji Hospital Affiliated Tongji Medical College, Huazhong University of Science and Technology, 1095 Jiefang avenue, Wuhan, 430030 Hubei China

**Keywords:** Baicalin, Hepatitis, Fatty liver disease, Xenobiotic-induced liver injury, Hepatocellular carcinoma, Liver ischemia reperfusion

## Abstract

Baicalin is the main active component of *Scutellaria baicalensis,* widely used in traditional Chinese medicine thanks to its various pharmacological effects, such as anti-tumor, anti-inflammatory, and antibacterial properties, as well as cardiovascular, hepatic, and renal protective effect. Recently, the protective effects of baicalin on liver disease have received much more attention. Several studies showed that baicalin protects against several types of liver diseases including viral hepatitis, fatty liver disease, xenobiotic induced liver injury, cholestatic liver injury, and hepatocellular carcinoma, with a variety of pharmacological mechanisms. A comprehensive understanding of the mechanism of baicalin can provide a valuable reference for its clinical use, but up to now, no narrative review is available that summarizes the pharmacological effects of baicalin to clarify its potential use in the treatment of liver diseases. Therefore, this review summarizes the progress of baicalin research and the underlying mechanism in the treatment of various liver diseases, to promote further research and its clinical application.

**Supplementary Information:**

The online version contains supplementary material available at 10.1007/s43440-021-00227-1.

## Introduction

Liver diseases are the main cause of illness and death worldwide [1, 2]. Approximately 25% of adults in the world suffer from non-alcoholic fatty liver disease, and approximately 75 million are diagnosed with alcohol-related disorders and are at risk of alcohol-associated liver disease [3]. The global prevalence of viral hepatitis remains high, while drug-induced liver injury continues to increase as the main cause of acute hepatitis. Approximately 1.16 million deaths and 788,000 deaths occur each year due to cirrhosis and liver cancer, respectively [3, 4]. Vaccination or newer drugs may reduce or relieve the burden of these liver diseases in developed countries, but the above remedies are still limited in developing countries [3]. Therefore, it is of utmost importance to find novel ways to effectively prevent the development and progress of liver diseases.

Baicalin possesses potential therapeutic effects on systemic diseases, including ocular disorders [5], periodontal diseases [6], inflammatory disorders [7], metabolic disorders [8] and even nervous system diseases [9, 10], thanks to its anti-inflammatory, anti-oxidative, anti-angiogenesis, and immunoregulatory effect. Moreover, baicalin possesses anti-obese, anti-viral, and anti-dyslipidemia effect, thus playing a critical role in improving liver function after injury or alleviating liver diseases [7, 11–13]. Since baicalin has a very wide range of pharmacological effects, more and more in-depth studies have been conducted. However, till now, no comprehensive review is available that summarizes the pharmacological effects of baicalin to clarify its potential use in the treatment of liver diseases. Therefore, the purpose of this review is to provide an update on the effects of baicalin and its mechanisms of action in the regulation of liver diseases.

## Plant source, isolation, and properties of baicalin

**Fig. 1:**
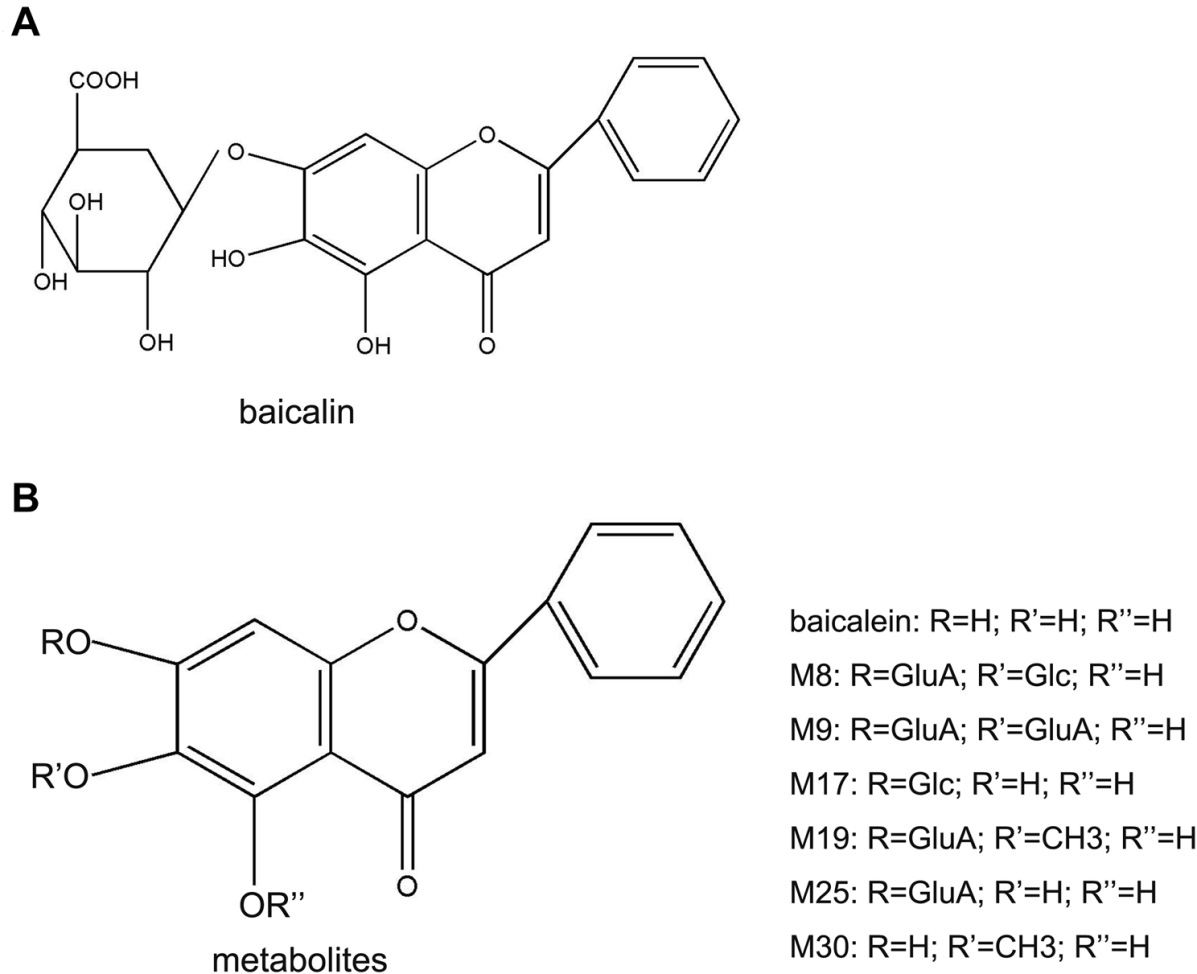
Structure of baicalin and main metabolites

Baicalin (C21H18O11; 5,6,7-trihydroxyflavone-7-β-D-glucuronide; Fig. 1a) is the main chemical and pharmacological component of Scutellaria baicalensis (SB) [14]. SB is often been called Huangqin or skullcap, is the dried root of *Scutellaria baicalensis Georgi*, and has been extensively used in the treatment of several diseases, including hepatitis, hyperlipidemia, dysentery, and liver cirrhosis in China and neighboring countries [15, 16]. *Scutellaria baicalensis Georgi* mainly grows in temperate regions and tropical mountains (with an altitude of approximately 1300–3000 m) in China, Russia’s Easter Siberia, Mongolia, North Korea, and Japan [17]. It is harvested in spring and autumn to remove the fibrous roots and sediments, then dried in the sun after removing the rough skin.

As the main flavonoid component, baicalin in SB is present at a concentration of at least 9.0%, making it as the quality standard of SB according to the Chinese Pharmacopoeia [18]. The conventional methods to purify baicalin from SB are solvent extraction and high-speed counter-current chromatography [19]. Nowadays, the present method to separate baicalin is more efficient and advantageous, with high efficiency and less solvent waste due to the advanced technology used [20].

As a flavonoid bearing glycoside, baicalin shows low water solubility (67.03 ± 1.60 μg/ml) and low permeability (*P*_app_ = 0.037 × 10^–6^ cm/m) [21], thus resulting in a low bioavailability after oral administration. The physicochemical properties of baicalin such as logP = 1.27, pKa1 = 7.6, pKa2 = 10.1, were determined at 25 °C in 0.1 M NaCl [22]. Ling et al. demonstrated that baicalin by itself is a poor radical scavenger (TEAC = 1.12, pH 7.4) and a poor antioxidant against free radical initiated lipid oxidation in liposomes, but this limitation can be reversed via the interaction with β-carotene [22]. In addition, γ-cyclodextrin complexation of baicalin can significantly increase its solubility by about 5 times. Thus, the physicochemical and structural properties of baicalin provide the data needed to design the dosage[23].

## The in vivo disposition of baicalin

A mutual transformation of baicalin and its aglycone, baicalein (Fig. 1b) occurs in the body once baicalin is absorbed. Indeed, baicalin is hydrolyzed to baicalein by β-glucuronidase in the intestine soon after administration, and then, baicalein is transformed back to baicalin by UDP-glucuronosyltransferase once it enters the systemic circulation [24]. Thus, baicalin is the major component in the systemic circulation rather than baicalein [25]. Baicalin concentration slightly increases in plasma (12–24 h) after oral administration, with a T_1/2TER_ of 12.1 h [26]. After *i.v.* administration, the elimination half-life (T_1/2_) and the terminal elimination half-life (T_1/2 TER_) of baicalin were 0.1 h and 9.7 h in plasma, respectively [26]. In addition, the peak plasma concentration of baicalin (C_max_, 0.91 ± 0.12 nmol/ml) is reached at 5 h after oral administration. According to the area under the curve (AUC), the absolute bioavailability (F%) of baicalin is 2.2 ± 0.2%, while one of its conjugated metabolites is 27.8 + 5.6% [26], indicating that bacalin is distributed in vivo mainly in the form of conjugates. The transport speed and distribution volume of baicalin depend on its binding degree with plasma proteins. The in vitro binding of baicalin to human serum proteins ranges from 86 to 92%, suggesting that baicalin may affect the effective concentration of other co-administered drugs [27]. Moreover, baicalin can pass the blood–brain barrier, thus it is able to exert protective effects against various neurodegenerative disorders [9, 10].

UDP-glucuronosyltransferase and β-glucuronidase are crucial metabolic enzymes involved in the biotransformation of baicalin. Indeed, five biliary metabolites were identified in the rat bile after oral administration of baicalin. Among them, the main metabolites are 6-o-β-glucopyranuronosyl-baicalein-7-o-sulfate and baicalein 6,7-di-o-β-glucupyranuroside [28]. These conjugated metabolites are then hydrolyzed to baicalein by β-glucuronidase/sulfatase in the gastrointestinal tract [28]. A total of 32 baicalin metabolites were identified by Zhang et al. in rat plasma, urine, and various tissues using more efficient approaches [29]. They found that baicalin in vivo undergoes several chemical transformations, such as methylation, hydrolysis, hydroxylation, methoxylation, glucuronide conjugation, sulfate conjugation, and their composite reactions. Five and 9 metabolites were found in rat liver and kidney, respectively, demonstrating that the rat liver and kidney are the most important organs for the distribution of baicalin after metabolism. In addition, six metabolites (M8, M9, M17, M19, M25, M30; Fig. 1c) may be related to the pharmacological effects of baicalin in vivo [29].

Baicalin is mainly excreted by the bile in the form of glucuronides and undergoes enterohepatic circulation [28]. Multidrug resistance-associated protein 2 is the main apical transporter of baicalin mediating the biliary efflux in the hepatocytes [30]. Indeed, the biliary excretion of baicalin is significantly decreased in multidrug resistance-associated protein 2 deficient rats, along with a markedly increased baicalin concentration in the plasma [31]. The fraction of baicalin and its conjugated metabolites is much less in the urine compared with the fraction in the bile [32]. The total urinary clearance of baicalein and baicalin is < 0.1%, while approximately 27% of baicalein is excreted in the stools as an unchanged drug [25].

The pharmacokinetics of baicalin can help the understanding of its pharmacological effects in the liver. The enterohepatic circulation ensures that baicalin is particularly concentrated in the liver, which is beneficial in the treatment of liver diseases. However, its metabolites such as baicalein also play a role in the treatment of liver diseases [33, 34], suggesting that the underline mechanisms of baicalin against liver diseases may involve the effect of its metabolites. Since baicalin is mainly metabolized in the liver, co-administration of baicalin with other herbs/drugs may affect its in vivo properties, ultimately affecting its effects. Therefore, the therapeutic dosage of baicalin should be carefully explored not only because of the above factors but also due to its low bioavailability, and techniques to enhance its absorption or liver targeting should be developed.

## Effect of baicalin on liver diseases

### Viruses hepatitis

Viral hepatitis is an infectious disease mainly caused by a variety of hepatitis viruses and represents a major health problem threatening human health worldwide. More than 240 million people are infected with HBV every year, and more than 80,000 patients die from HBV-related liver disease [35, 36]. Thus, it is of utmost importance to discover effective therapeutic drugs for its treatment.

Plant-derived flavonoids have certain advantages in combating viral infections, and their mechanisms of action include the prevention of virus invasion, inhibition of virus transcription, and its gene expression [37]. The flavonoid prescription containing baicalin exerts a significant curative effect in combating duck hepatitis A virus by the inhibition of its replication, the increase of T and B lymphocyte, the alleviation of liver damage, and the reduction of oxidative stress [38–40]. The combination of baicalin with catechin (both flavonoids) induces the cells to produce type I interferon (IFN-α/β) that significantly impairs the stability of HBV RNA, capsid, and cccDNA or reduced the amount of HBV replicative intermediates, transcripts, and HBV surface antigen levels [41]. Baicalin alone also improves the viability of duck embryonic hepatocytes infected by duck hepatitis A virus, reducing the virus reproduction in these hepatocytes and inhibiting the transcription of HBV RNA by down-regulating HNF-1α and HNF-4α expression [42, 43].

The combined application of baicalin and entecavir has a better good synergistic effect, better than the effect they exert when they are used alone since the combination exerts an enhanced virus suppression, induces a natural immune response, and controls HBV-related liver inflammation [43]. The synergistic effect may be due to the different targets of the two drugs, thus overcoming the obstacle of the nucleoside drugs currently used that only block HBV replication but they are ineffective in HBV cccDNA accumulation and HBV surface antigen loss. These results suggest that baicalin may be useful in the combination therapy against viral hepatitis.

### Fatty liver diseases (FLD)

The increased hepatic lipogenesis and serum non-esterified fatty acid lead to an excessive accumulation of lipids in the liver, causing fatty liver, impaired liver function, and eventually liver failure [44–46]. Baicalin is a potential therapeutic drug against FLD thanks to its several effects against it. Indeed, baicalin enhances lipid metabolism and represses hepatic de novo lipogenesis through the inhibition of the Ca2 + /CaM-dependent protein kinase β/AMP-activated protein kinase/acetyl-CoA carboxylase (CaMKKβ/AMPK/ACC) pathway [45, 47, 48]. In addition, baicalin directly binds carnitine palmitoyltransferase 1, consequently promoting the lipid influx into mitochondria where they are oxidated [49], down-regulating lipogenesis genes such as sterol regulatory element-binding protein-1c, fatty acid synthase, and peroxisome proliferator-activated receptor alpha to reduce hepatic lipid accumulation [46, 47, 50].

Another ability of baicalin is to suppress liver fibrosis, systemic inflammation, and oxidative stress in FLD. Indeed, it inhibits the expression of α-smooth muscle actin, transforming growth factor beta 1 and collagen type I alpha 1 chain to reverse liver fibrosis [44–46]. Furthermore, it reverses the epithelial‑mesenchymal transition through the inhibition of the TGF‑β1/Smad3 pathway in vitro to prevent the development of liver fibrosis [51]. In addition, baicalin exerts an indirect antioxidant function by upregulating hepatic glutathione and superoxide dismutase levels and downregulating malondialdehyde levels in mice subjected to a high fat and high cholesterol diet [44]. Moreover, baicalin reduces systemic inflammation by blocking NLR pyrin domain containing 3–gasdermin D signaling, as demonstrated by in vitro experiments, and by the decreased release of pro-inflammatory factors via the inhibition of toll-like receptor 4 (TLR4) signaling cascade in mice [44, 46, 52–54].

Finally, baicalin alleviates glucose intolerance, hyperglycemia, and insulin resistance in mice under a diet inducing obesity. Baicalin significantly decreases insulin concentrations due to a high-fat diet, and the underlying mechanisms are related to the inhibitory effect of baicalin on the expression of p-p38, MAPK, p-CREB, FoxO1, PGC-1α, PEPCK and G6Pase [45, 47, 55]. Thus, baicalin may prevent FLD thanks to its multiple pharmacological effects.

### Xenobiotic-induced liver injury

Baicalin exerts potential benefits on liver injury induced by some xenobiotics, such as drugs and environmental toxins. It significantly attenuates oxidative stress and inflammatory response in acetaminophen (APAP)-treated mice [56–58]. Besides, baicalin stimulates the detoxification of APAP by preventing APAP-induced depletion of glutathione and suppresses CYP2E1 activity, the latter responsible for the production of N-acetyl-p-benzoquinoneimine, a cytotoxic APAP intermediate metabolite [59].

LPS-induced liver injury is mainly related to excessive inflammation in the liver. Yanqiu et al. [60] found that baicalin decreases cell apoptosis and inflammatory reaction due to LPS through the upregulation of taurine upregulated gene 1 and the inactivation of both p38MAPK and Jun N-terminal kinase pathways, as well as through the reduction of NF-κB translocation. The underlying mechanism is associated to the up-regulation of the activity and expression of heme oxygenase 1 [61].

Iron overload is one of the most common causes of metal-related liver toxicity. It leads to a significant increase in serum ALT and AST levels and SOD activity, and a decrease in the sulfhydryl content and glutathione peroxidase activity [62]. Baicalin not only alleviates hepatic damage due to iron accumulation, but also reverses the above abnormal serum indicators and the nitration level, and reduces serum iron content [63, 64]. Baicalin also ameliorates cadmium chloride-induced hepatic cytotoxicity and confers protection against CCl4-induced liver injury through the reduction of the inflammatory response, hepatic fibrosis, and oxidative damage [65–68]. Similarly, baicalin ameliorates ethanol-induced liver injury through the modulation of hepatic oxidative stress, inflammation, and Sonic Hedgehog signaling pathway in rats [69, 70]. Consequently, baicalin shows great therapeutic potential against various xenobiotics that induce liver injury, thus deserving further exploration.

### Hepatocellular carcinoma (HCC)

HCC causes the death of more than 600,000 people each year and has become the third leading cause of cancer-related death worldwide [71]. Less than 30% of patients with HCC have the opportunity to be subjected to surgery due to their poor physical condition, thus, the 5 year survival rate of these patients is only 9% [71, 72].

Baicalin shows a potential therapeutic effect against HCC through the induction of cell apoptosis and autophagy in the tumor. Baicalin inhibits HCC cell viability and proliferation in vitro by down-regulating the expression of cyclin A, cyclin-dependent-kinase 2 and cyclin D1 and inducing apoptosis [73, 74], while it significantly inhibits the growth of xenografts in vivo in nude mice[73]. In addition, Baicalin induces cell apoptosis in HepG2 and SMMC-7721 cells through the up-regulation of Bax, the down-regulation of Bcl-2, the induction of the cleavage of Caspase-9 and Caspase-3, and the induction of poly ADP-ribose polymerase [73]. The underlying mechanism may be potentially related to the ability of baicalin to activate the transcription factor 6 signaling pathway through targeting the site-2 protease in HCC cells [74]. Furthermore, baicalin suppresses the growth of HCC by inducing cell autophagy via the regulation of tumor-associated macrophages repolarization [75]. Despite all these discoveries, the knowledge of the pharmacological mechanisms used by baicalin to regulate HCC inhibition is still limited, and further studies are needed to provide additional evidence for a potential application of baicalin as alternative medicine in HCC treatment.

### Liver ischemia reperfusion (IR) injury

Liver IR occurs in several clinical circumstances, including liver transplantation, partial hepatic resection, shock, and hepatic failure [76, 77]. IR injury begins with the onset of hypoxia, which causes cellular damage, followed by the restoration of the blood flow and oxygen delivery, which exacerbates cellular damage [78, 79]. At present, no effective prevention strategy is available against liver IR injury.

Baicalin attenuates oxidative damage, increases cell viability and decreases the level of lactate dehydrogenase [80], suggesting a potential protective effect on hepatic IR injury. Indeed, baicalin significantly increases HO-1 expression and reduces inducible nitric oxide synthase, cyclooxygenase-2, TNF receptor 1-associated protein expression, and toll-like receptor 4-mediated inflammatory response in liver IR animals [81, 82]. Moreover, baicalin inhibits the mitochondrial swelling rate and caspases-3 and 8 activation in IR rats [82]. Furthermore, baicalin exerts a protective effect on liver IR injury through the induction of cell autophagy, but the detailed mechanisms regulating autophagy in liver IR injury should be further investigated by both i*n vivo* and in vitro experiments [80].

### Cholestatic liver injury

Cholestasis is a bile flow disorder caused by various factors, and it is characterized by an accumulation of toxic bile acids in the liver, causing the damage of hepatocytes and bile ducts and eventually liver fibrosis or liver failure [83]. Thus, an acceleration in the flow and excretion of bile acids and their reduced accumulation are the key points to consider in the development of a drug to combat cholestasis [84]. Co-administration of the *Scutellaria baicalensis* extract and metformin promotes the conversion of cholesterol to bile acids and accelerates their fecal excretion [85]. Shen et al. found that baicalin administration in mice significantly alleviates intrahepatic cholestasis and fibrosis after bile duct ligation [86]. Moreover, our previous work demonstrated that baicalin exerts a protective effect on estrogen-induced cholestatic liver injury via the induction of the sirtuin 1/hepatic nuclear receptor-1a/FXR signaling pathway, consequently maintaining hepatic bile acid homeostasis and alleviating hepatic inflammation [87, 88]. However, the protective effect of baicalin on cholestatic liver injury needs further investigation in the future due to the complex pathogenesis of this injury, and multi-omics technology might be used to improve our knowledge on this protective effect.

## Liver targeting strategy of baicalin

Although baicalin exhibits many biological activities against liver disease, its low lipid and water solubility limits its clinical application. To be more effective against liver diseases, the drugs should specifically target the liver, thus, the clinical application of baicalin should also meet this aspect. This is why different baicalin-based formulations and drug delivery systems have been studied in order to improve the absorption and bioavailability of baicalin and enhance its effect on liver disease. For example, the baicalin-loaded liposome formulation improves the lipophilicity of baicalin and further enhances its concentration in the liver [89, 90]. Indeed, this formulation exerts a greater effect in the treatment of non-alcoholic fatty liver disease than baicalin alone [54]. In addition, Iman et al. [74, 91] used lactobionic acid-modified-chitosan lactate polymer to obtain a site-specific baicalin delivery to increase the liver targeting ability of this drug. As expected, the concentration of baicalin in the liver after oral administration was remarkably increased and the mean area under the curve (AUC_0–24_) was much higher after the use of this polymer as a delivery system. These strategies show great potential in the increase of the bioavailability of baicalin in the liver after oral administration.

## Advantages and safety of baicalin in liver diseases

**Fig. 2:**
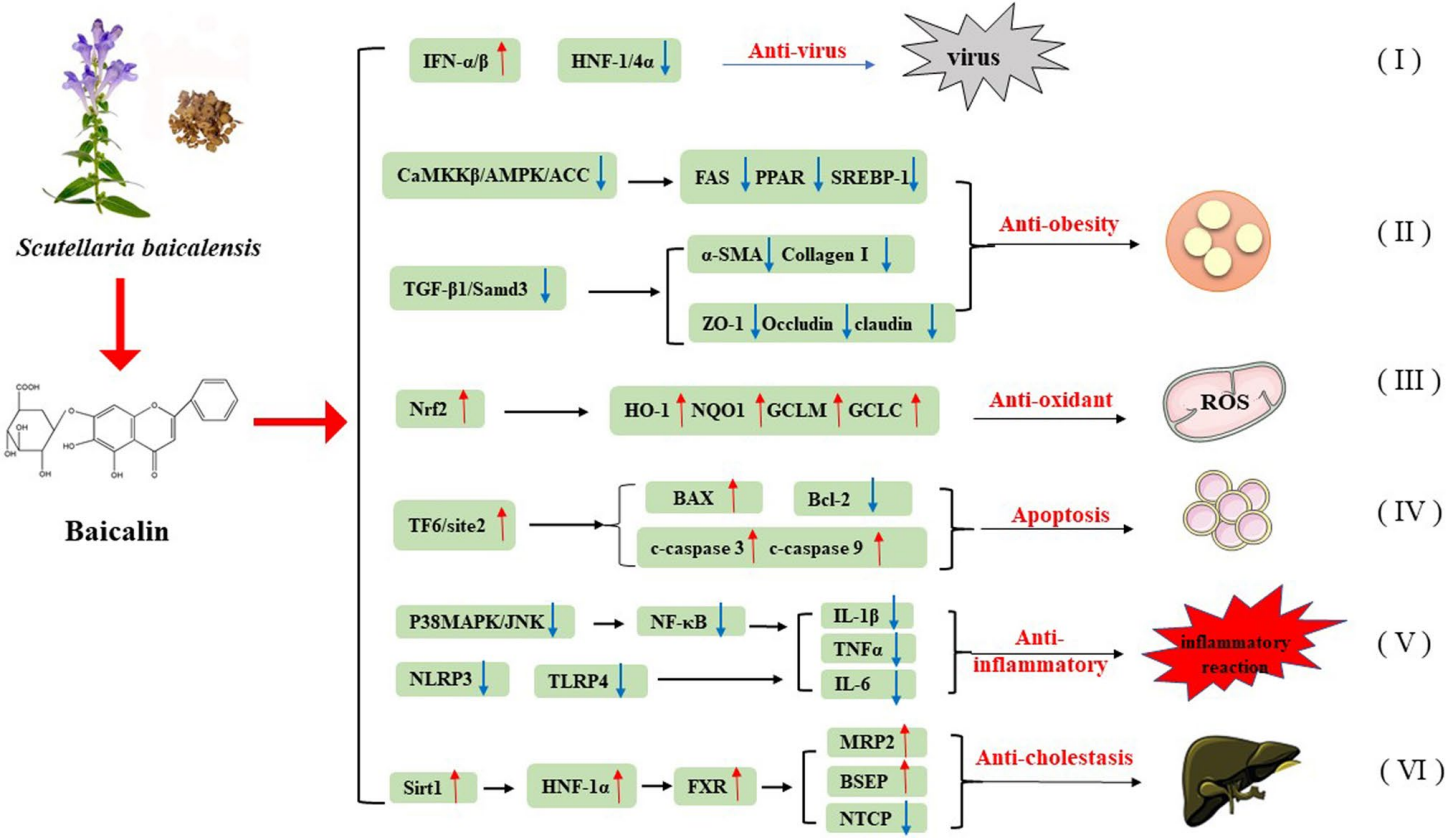
Main pharmacological mechanisms of baicalin on liver diseases: (I) Baicalin induces the production of IFN-α/β and down-regulates the expression of HNF-1/4α to reduce the replication of HBV; (II) Baicalin inhibits the CaMKKβ/AMPK/ACC and TGF‑β1/Smad3 signaling pathway, thus represses hepatic de novo lipogenesis and prevents the development of liver fibrosis; (III) baicalin activates the transcription factor 6 signaling pathway through targeting the site-2 protease to induce cell apoptosis in HCC cells; IV, baicalin increases the Nrf2/HO-1 signaling pathway to attenuate oxidative damage; V, baicalin decreases the release of pro-inflammatory factors by blocking NLRP3 or TLRP4 or p38MAPK/JNK/NF-κB pathways and improves systemic inflammation; (VI) baicalin maintains hepatic bile acid homeostasis via the induction of the sirt1/HNF-1α/FXR signaling pathway. The red arrows indicate the pathways which are up-regulated, and the blue arrows mean the pathways that are down-regulated by baicalin

Clinical management of liver diseases is still rather challenging due to late diagnosis, progressive characteristic, therapeutic drugs with a poor effect, and limited therapeutic strategies [1, 3]. Several drugs are commonly used in the treatment of liver diseases, such as ursodeoxycholic acid [92], cholestyramine [93], corticosteroids [94], and simvastatin [95], but with barely satisfactory clinical results. However, an increasing number of active molecules and small molecule inhibitors have been found to be hepatoprotective [96–99]. Most of them are poorly studied and the underline mechanisms and toxic effects are still unclear. Baicalin is derived from SB, which has been extensively used throughout China’s long history to combat liver diseases, and baicalin itself has also been applied in clinical practice for decades [14, 16]. Distinguished from the above hepatic active molecules, baicalin presents diversified pharmacological action and hepatoprotective effects, such as anti-viral, anti-oxidant, anti-inflammatory and anti-obesity effects, resulting a potential candidate against liver diseases (Supplementary Table 1; Fig. 2.).

A study showed that baicalin may have potential nephrotoxic effects in rats at high doses (800‒1600 mg/kg), although no toxic to the liver of rats [100]. At the administered dose, no evident toxic and side effects of baicalin or its aglycone baicalein were observed in many clinical studies. A study showed that a single oral dose of 100–2800 mg baicalein is safe and well-tolerated by healthy subjects, with no signs of toxicity in the liver or kidney [25]. Besides, the capsules of baicalin have been applied in clinical practice as an adjuvant treatment of acute and chronic hepatitis and evasive hepatitis. Zhang et al. found no abnormal behavior in ICR mice at the maximum dose of capsules containing 15 g/kg baicalin after oral administration. Moreover, no death occurred among the mice within one week of administration, and no abnormalities were observed in any organ after dissection. These results demonstrated that baicalin capsules possess a good safety profile [101]. Thus, the favorable safety profile of baicalin merits further studies for its clinical use. Overall, baicalin may be a promising therapeutic option in the treatment of liver diseases.

## Conclusion

Liver diseases represent a significant burden of disease and death worldwide. The development of liver disease is related to a variety of factors such as alcohol, drugs, viruses, and obesity, and the mechanisms involved are several, including inflammation, oxidative stress, liver fibrosis, and apoptosis. Currently available drugs are not ideal for treating liver diseases, thus, new drugs are still needed. SB has been widely used for thousands of years in the treatment of liver and gallbladder diseases. Baicalin is the main chemical component and the primary pharmacologically active compound of SB. This article summarizes the wide range of pharmacological properties of baicalin, such as antioxidant, antiviral, anti-inflammatory, anti-obesity, and anti-tumor effects, thus it might be of great value in combating liver diseases (Supplementary Table 1; Fig. 2). A comprehensive understanding of the mechanism of action of baicalin might provide a valuable basis and reference for its clinical use. Nevertheless, extensive studies are still needed to discover its key targets, and clarify its clinical indications. Indeed, the knowledge of baicalin mechanism of action is still limited to animals and in vitro studies, and it is still necessary to further explore the underlying mechanisms to verify its effects and safety in clinical trials. Besides, the low hydrophilic property and poor absorption of baicalin limit its application. Thus, new technologies and new preparations are urgently needed and should be developed to improve its solubility and liver targeting ability. Although baicalin is potentially effective in the management of several liver diseases described in this review, its role in autoimmune liver disease is scarcely reported. Therefore, more basic and applied studies should be performed to comprehensively clarify the pharmacological mechanisms and application of baicalin in the treatment of liver diseases.

## Supplementary Information

Below is the link to the electronic supplementary material.

Supplementary file1 (DOCX 21 KB)
