# Supplementary material for: Pharmacological properties of baicalin on liver diseases: a narrative review
Source: Pharmacol Rep. 2021 Feb 17;73(5):1230–9. doi: 10.1007/s43440-021-00227-1 (PMC8460515; doi:10.1007/s43440-021-00227-1)
Supplement: Supplementary file 1 — Supplementary file1 (DOCX 21 KB) [file 43440_2021_227_MOESM1_ESM.docx]

**Pharmacological properties of baicalin on liver diseases: a systematic review**

Jin-yu Yang, Min Li, Cheng-liang Zhang*, Dong Liu*

**Jin-yu Yang,** Department of Pharmacy, Tongji Hospital Affiliated Tongji Medical College, Huazhong University of Science and Technology, Wuhan, China

**Min Li,** Department of Pharmacy, Tongji Hospital Affiliated Tongji Medical College, Huazhong University of Science and Technology, Wuhan, China

**Cheng-liang Zhang,** Department of Pharmacy, Tongji Hospital Affiliated Tongji Medical College, Huazhong University of Science and Technology, Wuhan, China

**Dong Liu,** Department of Pharmacy, Tongji Hospital Affiliated Tongji Medical College, Huazhong University of Science and Technology, Wuhan, China

**Corresponding author: Cheng-liang Zhang,** PhD, Associate chief pharmacist, Associate Professor, 1095 Jiefang Avenue，Department of Pharmacy, Tongji Hospital Affiliated Tongji Medical College, Huazhong University of Science and Technology, 1095 Jiefang avenue, Wuhan, Hubei 430030 P.R of China. [clzhang@tjh.tjmu.edu.cn](mailto:clzhang@tjh.tjmu.edu.cn). **Dong Liu,** PhD, Chief pharmacist, Professor, Department of Pharmacy, Tongji Hospital Affiliated Tongji Medical College, Huazhong University of Science and Technology, 1095 Jiefang avenue, Wuhan, Hubei 430030 P.R of China. [ld2069@outlook.com](mailto:ld2069@outlook.com).

Supplementary Table 1

**Reports on the pharmacological roles of baicalin on liver diseases**

| **Disease** | **Etiology** | **Model** | **Dose(baicalin)** | **Mechanism** | **Reference** |
| --- | --- | --- | --- | --- | --- |
| **Hepatitis** | - DVH - HBV | - HuH7 and HepG2 with HBV - Duck with DVH | - 25, 50, 75, or 100 μM - Flavocoxid (25, 75, 100, 125, and 250 µg/ml) | - Reduces inflammation and oxidative - Inhibits the replication of HBV | [38-43] |
| **Fatty liver diseases** | - High fat diet - High cholesterol diet - FFA - Sugar - OA - PA | - Mice with high fat/ high cholesterol / OA/MCD diet - KK-A^y^ mice - HepG2 or AML-12 cells with FFA/sugar/PA | - HFC diet containing 0.5% w/w baicalin - 50,100,200,400 mg/kg | - enhances lipid metabolism and represses hepatic de novo lipogenesis - suppress liver fibrosis, systemic inflammation and oxidative stress - Alleviates glucose intolerance, hyperglycemia and insulin resistance | [45-54] |
| **Xenobiotic-induced liver injury** | - APAP - LPS - Iron - Cd - CCl4 - alcohol | - Mice with APAP(300 mg/kg) - iron overload rats (500 mg iron/kg) - Rats with Cd (6.5 mg/kg body weight) - L-02 and THLE2 cell with LPS - L-02 with alcohol | - 0, 15, 30, or 60 mg/kg - baicalin-containing diet (0.3%, 0.25% and 1% w/w) - 10, 20, 30, 40, 50 mg/kg; - 20,50 μM | - Decreases hepatic inflammatory responses - Increases the detoxification of APAP - Antioxidation - Anti-apoptosis - Anti-fibrosis | [56-70] |
| **Hepatocellular carcinoma** | - SMMC-7721 - Hep G2 | - athymic nude mice injected with Hep G2 cells | - 0, 10, 20, and 40 μmol/L - 50 mg/kg | - induces cell apoptosis - induces cell autophagy | [73-75] |
| **Liver ischemia reperfusion** | - I/R-induced liver injury - alcohol | - rats were subjected to ischemia followed by reperfusion - Rats with alcohol liquid diet - LO2 cells undergo Hypoxia/reoxygenation (H/R) | - 200 mg/kg - 50, 100, 200 µmol/l | - Reduces inflammation - Improves oxidative stress - Decreases cell apoptosis | [80-82] |
| **Cholestatic liver injury** | - BDL - 17α-ethinylestradiol (EE) | - BDL mice - SD rats treated with EE (5 mg/kg) | - 50,100,200 mg/kg | - Inhibits hepatic inflammatory response - Reduces liver fibrosis - Enhances antioxidant - Maintains hepatic BA homeostasis | [85-88] |

Flavocoxid: FDA-regulated medical food containing the naturally occurring flavonoids, such as baicalin extracted from Scutellaria baicalensis, and catechin from Acacia catechu.

I/R: cells were seeded in 6-well plates at a density of 10^5^ cells/well. When the cells reached 80% confluence, the 6-well plates were incubated under hypoxic conditions (94% N_2_, 5% CO_2_ and 1% O_2_ at 37 ˚C) for 24 h in a hypoxia chamber.
